# Experimental investigation of indoor aerosol dispersion and accumulation in the context of COVID-19: Effects of masks and ventilation

**DOI:** 10.1063/5.0057100

**Published:** 2021-07-21

**Authors:** Yash Shah, John W. Kurelek, Sean D. Peterson, Serhiy Yarusevych

**Affiliations:** Mechanical and Mechatronics Engineering, University of Waterloo, Waterloo, Ontario N2L 3G1, Canada

## Abstract

The ongoing COVID-19 pandemic has highlighted the importance of aerosol dispersion in disease transmission in indoor environments. The present study experimentally investigates the dispersion and build-up of an exhaled aerosol modeled with polydisperse microscopic particles (approximately 1 *μ*m mean diameter) by a seated manikin in a relatively large indoor environment. The aims are to offer quantitative insight into the effect of common face masks and ventilation/air purification, and to provide relevant experimental metrics for modeling and risk assessment. Measurements demonstrate that all tested masks provide protection in the immediate vicinity of the host primarily through the redirection and reduction of expiratory momentum. However, leakages are observed to result in notable decreases in mask efficiency relative to the ideal filtration efficiency of the mask material, even in the case of high-efficiency masks, such as the R95 or KN95. Tests conducted in the far field ( 2 m distance from the subject) capture significant aerosol build-up in the indoor space over a long duration ( 10 h). A quantitative measure of apparent exhalation filtration efficiency is provided based on experimental data assimilation to a simplified model. The results demonstrate that the apparent exhalation filtration efficiency is significantly lower than the ideal filtration efficiency of the mask material. Nevertheless, high-efficiency masks, such as the KN95, still offer substantially higher apparent filtration efficiencies (60% and 46% for R95 and KN95 masks, respectively) than the more commonly used cloth (10%) and surgical masks (12%), and therefore are still the recommended choice in mitigating airborne disease transmission indoors. The results also suggest that, while higher ventilation capacities are required to fully mitigate aerosol build-up, even relatively low air-change rates ( $2\;h^{-1}$) lead to lower aerosol build-up compared to the best performing mask in an unventilated space.

## I. INTRODUCTION

Expiratory events, such as breathing, speaking, sneezing, or coughing, produce droplets, ranging in micrometers to millimeters in size, that serve as the primary pathway for the transmission of many infectious diseases, including coronavirus disease 2019 (COVID-19).^1–6^ The ongoing COVID-19 pandemic underscored glaring gaps in our understanding of pathogen transmission required to effectively contain and prevent outbreaks, including, but not limited to, the development of reliable guidelines for safe social distancing,^1,2^ usage of personal protective equipment (PPE),^7,8^ and indoor ventilation.^1,9^ The initial guidelines released in early 2020 by the World Health Organization and many national health agencies assumed that COVID-19 spreads primarily through large droplets that settle on surfaces within 1 to 2 m from the infected individuals. Although an intense scientific debate on the main transmission pathways of COVID-19 continues,^10,11^ the mounting data on local outbreaks and relevant research^9,12,13^ have prompted significant modifications to official guidelines, which now attribute the spread of COVID-19 to a wide range of droplet sizes, including both larger respiratory droplets and microscopic aerosols, produced during various expiratory events.^14–16^

Recent research has shown that smaller droplets and droplet nuclei containing significant viral load can travel up to 8 m during expiratory events,^3^ substantially exceeding the present social distancing limits. Furthermore, the severe acute respiratory syndrome coronavirus 2 (SARS-CoV-2) has been shown to retain infectivity in aerosol form for a minimum of three hours past expiration from an infected person,^5^ making it possible for the pathogens to be transported over extended distances due to ambient flows in indoor environments.^6,11,17–20^ The critical importance of safety considerations at indoor workplaces has been recently highlighted by the analysis of various COVID-19 superspreader events,^13^ the vast majority of which took place indoors. This underscores the importance of understanding the spread and accumulation of human-borne aerosols in indoor environments through the lens of social distancing, mask usage, occupancy, exposure duration limits, and ventilation.

Another contentious issue brought to the forefront of scientific debates by the COVID-19 pandemic is the efficacy of face masks.^7,8^ Although some clinical evidence of respirator mask efficiency existed prior to the COVID-19 pandemic,^21^ there is mounting evidence that continuous usage of appropriate face masks can reduce the rate of virus transmission;^7,8^ however, the efficacy of different mask types for both the reduction of viral emissions and prevention of individual inhalation of pathogens requires further quantitative analysis.^1,2^ This is of particular importance for indoor settings, where PPE can significantly affect the accumulation of the pathogens and their transmission through filtration and reduction/redirection of momentum during expiratory events.

Recent observational studies and meta-analyses of mask effectiveness have estimated that mask usage reduces the risk of respiratory virus spread by 70% to 80%.^22^ Efficacy of home-made masks at preventing spread of influenza showed that surgical masks are three times more effective at blocking micro-organism transmission than home-made masks^23–25^ although none of these studies include randomized control trials.^26^ There is, however, evidence that communities in which masks were in widespread use exhibited significantly reduced community spread.^24,25^

The higher risk of infectious disease transmission in indoor environments, particularly with poor ventilation, has been recognized in the scientific community well before the onset of the COVID-19 pandemic^27^ and prompted a number of studies on transmission and aerosol transport indoors.^28–31^ The ongoing COVID-19 pandemic has re-invigorated the research efforts in this area due to the growing association between local outbreaks and various indoor settings. For example, Qian *et al.*^13^ reported that all except one of the 318 analyzed COVID-19 outbreaks were associated with indoor spaces. Bhagat *et al.*^32^ provided an overview of potential effects of ventilation on the indoor spread of COVID-19, and general guidelines for minimizing airborne transmission are detailed in Morawska *et al.*^11^ Mittal, Meneveau, and Wu^33^ proposed a framework for estimating the risk of airborne transmission of COVID-19 based on probabilistic factors and highlighted the critical need for reliable quantification of key model parameters for future modeling and validation.

The airborne transmission of pathogens in indoor environment is directly related to the dynamics of virus-laden aerosols.^28^ The associated transmission risk models are based on either simplified analytical formulations^34–36^ or computational fluid dynamics (CFD) tools.^17,33,37^ The former typically employ a well-mixed room assumption, where pathogen carrying aerosols are assumed to be instantaneously and uniformly distributed in a given room, such as in the classical Wells–Riley equation.^34^ Such simplified modeling has also been employed for COVID-19 risk assessment.^36,38^ On the other hand, at the expense of notably higher computational costs and model complexity, CFD-based modeling can provide added insight into spatial evolution of aerosols produced by various expiratory events in realistic indoor environments. Along these lines, a number of studies have modeled airborne spread of COVID-19;^17,39^ however, all models rely on quantitative results from experimental studies for an array of input parameters, such as the initial number and size distribution of aerosol particles and initial velocities and duration of expiratory events. Further, the use of PPE, including face masks, needs to be incorporated into computational models, which either significantly complicates the modeling^40^ or requires experimental data.^33^ Thus, there is a need to incorporate the progress made in a number of recent qualitative and quantitative studies focused on PPE performance into larger-scale investigations focused on aerosol dispersion in indoor environments. This will provide a more comprehensive outlook on workplace health and safety, where the use of PPE is not only often mandated by local legislature, but also can help mitigate the limitation of available ventilation options.

The present study is aimed at bridging the gap between studies focused on face mask efficacy assessment and indoor dispersion of aerosols by experimentally evaluating the aerosol accumulation in a controlled indoor environment, with various types of face masks and ventilation settings considered. Typical nasal breathing is modeled in the present work using a high-fidelity physical model. While this type of expiration is known to produce the lowest aerosol counts per event, this type of breathing is the most common type of expiration and thus accounts for the majority of aerosol production during continuous occupancy at work and public places.^41^ A combination of flow visualization, velocity and concentration measurements, and modeling is used to provide a quantitative outlook on the effect of different face masks on aerosol build-up over extended time periods in a generic indoor setting. The result provides critical estimates of apparent filtration efficiency essential for producing adaptive health and safety guidelines for workplaces during pandemic and epidemic events as well as for the development of advanced modeling tools.

## II. METHODOLOGY

**FIG. 1.**
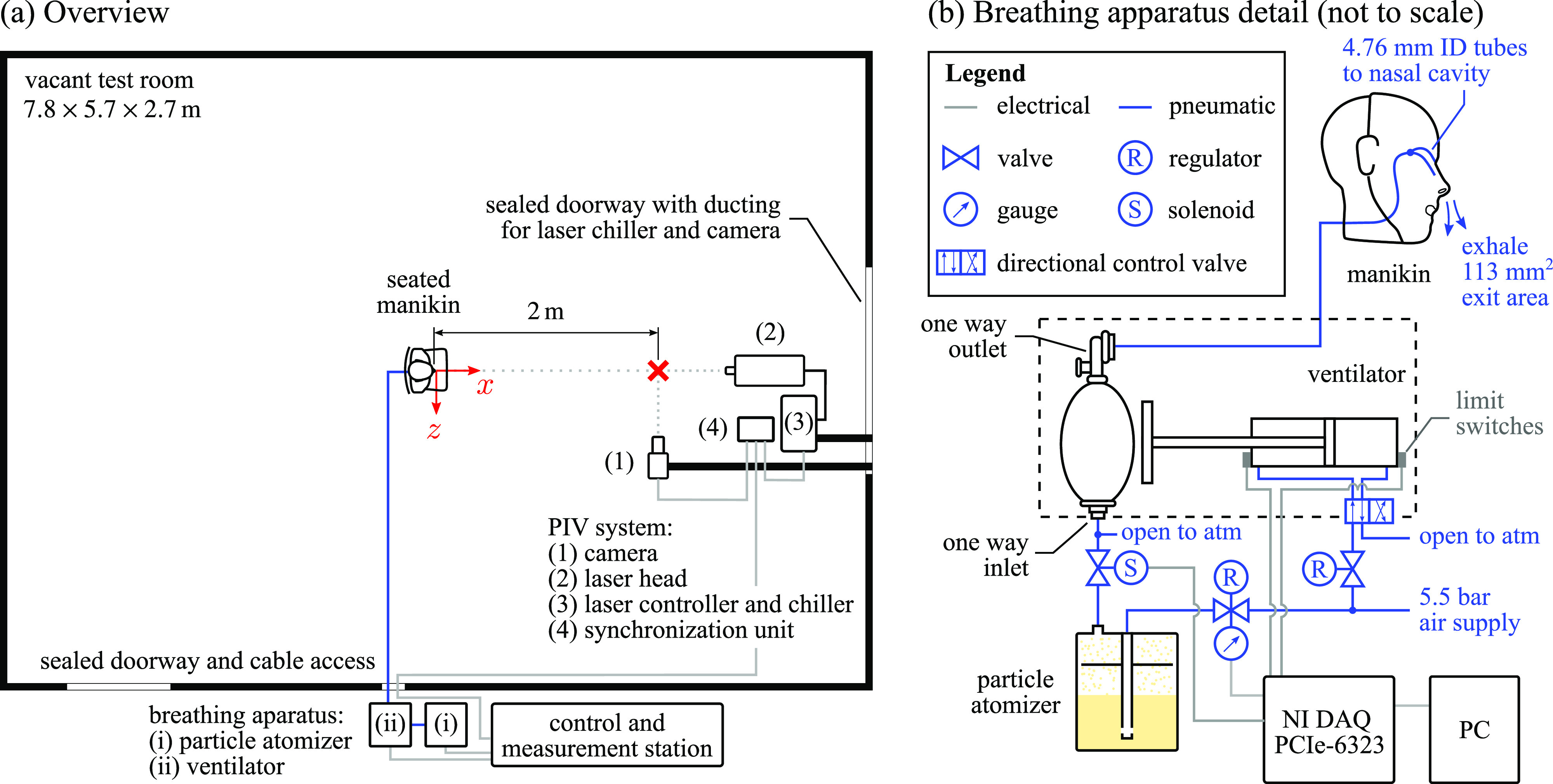
(a) Overview of the experimental setup and (b) details of the breathing apparatus.

Experiments were conducted in the Fluid Mechanics Research Laboratory at the University of Waterloo. An overview of the setup is provided in Fig. 1(a). All tests were performed in a 7.8×5.7×2.7 m room with an air volume of approximately $120\;m^{3}$ that was vacated except for the test model and essential equipment. To study the dispersion of exhaled aerosols in an unventilated space, the room was sealed from all surroundings, which included shutting off the ventilation system and sealing all air passageways through the room envelope.

**FIG. 2.**
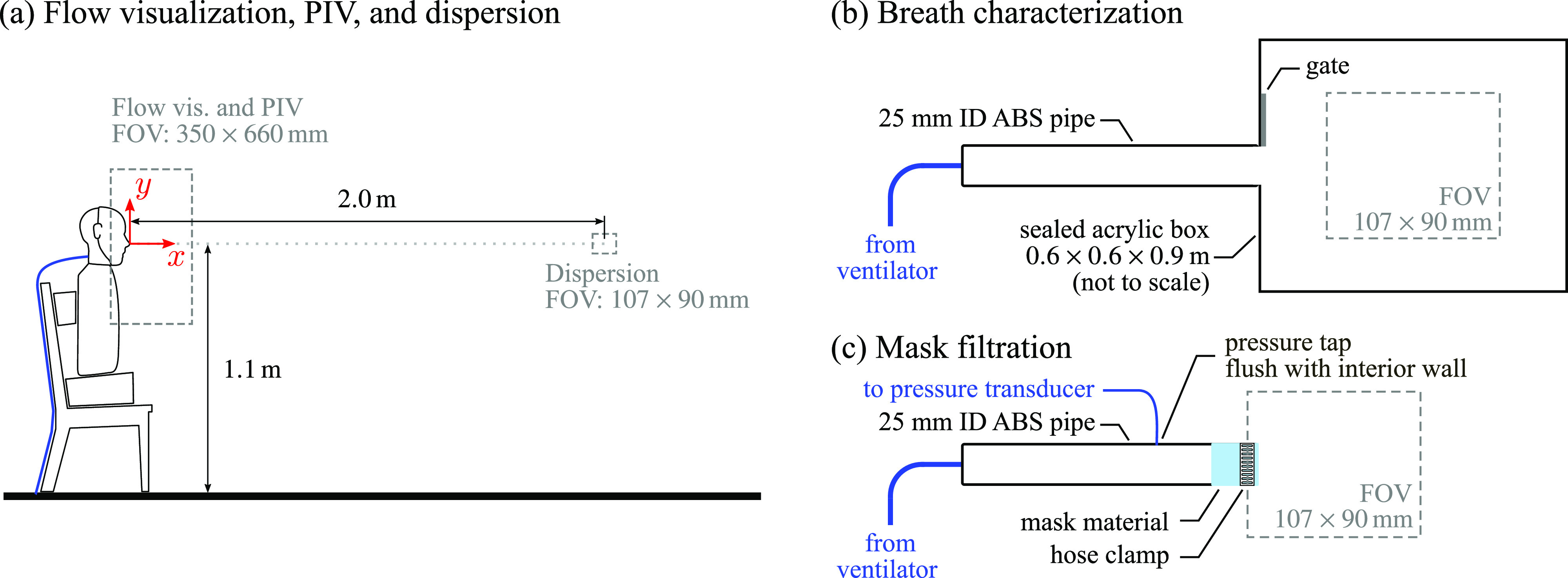
Profile view of experimental setups for (a) flow visualization, PIV, and dispersion tests, (b) breath characterization, and (c) mask filtration tests.

The test model was a Prestan adult CPR manikin (model PP-AM-100-DS), placed upright in a seated position on a chair in the center of the room [Figs. 1(a) and 2(a)]. Breathing with aerosol-laden exhalation was provided by a custom breathing apparatus, the details of which are provided in Fig. 1(b). The positive and negative air pressure cycle was provided by a mechanical ventilator (developed and donated to the project by Crystal Fountains, Inc.), which operated through the repeated compression and decompression of an adult size med-rescuer bag-valve-mask (BVM) (1500 mL bag volume) by a pneumatic piston. Physiological parameters representative of typical adult nasal breathing^42^ were set in terms of respiratory period/rate, exhalation time, and breath volume through adjustment of the piston plunging depth, forward and backward stroke speeds, and contact time with the BVM, resulting in the breathing parameters reported in Table I. These parameters were monitored and logged during operation of the ventilator via forward and backward stroke limit switches on the piston, with signal sampling performed using National Instruments' LabVIEW software and a PCIe-6323 DAQ.

**TABLE I.** Breathing and aerosol production parameters (95% confidence).

Parameter	Value	Unit
Exhalation time	1.75±2%	s
Respiratory period	4.32±2%	s
Respiratory rate	13.9±2%	breath/min
Breath volume	690±3.5%	mL
Avg. exhalation flow rate^a^	0.40±4%	L s^−1^
Particles injected	$2.75\times 10^{8}\pm 7.5\%$	particles/breath
Breath particle concentration	$3.98\times 10^{8}\pm 8\%$	particles/l

^a^ Flow rate computed over exhalation time.

Aerosols were produced by atomizing olive oil into particles with a mean diameter of about 1 *μ*m (volume-weighted) using a Laskin nozzle style atomizer based on the designs of Kahler, Sammler, and Kompenhans.^43^ Controlled injection of particles into the breathing stream was achieved using a normally closed solenoid valve located downstream of the atomizer, the opening of which was synchronized with the breathing cycle through LabVIEW. The solenoid was opened at the start of inhalation (i.e., at the forward stroke limit) and was held open for 2.0 s as this matched the re-inflation time of the BVM without particle injection. The particle production rate was controlled by a pressure regulating valve upstream of the atomizer, the pressure of which was logged in LabVIEW and remained within 0.172 bar±0.5 % throughout operation. This pressure level was verified to be below the minimum pressure needed to open the BVM outlet valve, therefore ensuring exhaust from the BVM during the exhalation (compression) portion of the cycle only. Olive oil was selected for the aerosol liquid component as its use in an atomizer of this design is known to give a polydisperse distribution of particle sizes with a mean diameter of approximately 1 *μ*m,^43^ matching the smaller scale of aerosols expelled during typical human respiration,^41,44,45^ and the nuclei formed after larger droplets evaporate.^46^ Furthermore, oil-based aerosols offer good light scattering properties for optical detection, are charge-neutral,^47^ and have a residence time on the order of several hours, in comparison to several minutes for water-based alternatives, which is critical given the approximate one hour viability half-life of the SARS-CoV-2 virus^5^ and that virion containing droplet nuclei may remain suspended in air for hours.^2^ It should be noted that higher exhalation aerosol concentrations than those typical for normal breathing^44,48,49^ was employed (Table I) to reduce measurement uncertainty in experimental aerosol concentration estimates. For consistent data comparison, all concentration measurements are normalized by the initial breath particle concentration, which was maintained constant throughout the study.

The breathing apparatus and measurement control station were located outside of the test room [Fig. 1(a)], with all connections to the interior passed through a sealed cable access. This allowed for tests to be controlled from outside the room, thereby removing any unintended effects the presence of the breathing apparatus, control and measurement equipment, and/or test operators may have had on the results. The output of the ventilator was connected to the manikin using a 12.7 mm inner diameter flexible tube, which was split into two 4.76 mm inner diameter tubes that exhaust into the manikin's nasal cavity. The total nostril exit area was $113\;{\text{ms}}^{-1}$, which is within the expected range for male adults.^50^ The limitations of this experimental setup are the absence of a thermal plume typically present around a human being^32^ and that the inhalation does not occur at the manikin, but rather at the inlet of the BVM [Fig. 1(b)].

Qualitative and quantitative measures of aerosol dispersion from the test model were performed using simultaneous illumination and imaging of particles with a laser and digital camera, respectively [Fig. 1(a)]. The methodology constitutes planar particle image velocimetry (PIV) measurements,^51^ with the specific equipment employed including an EverGreen 70 mJ/pulse Nd:YAG laser, PCO sCMOS cameras (5.5 MP, 6.5 *μ*m pixel pitch) fitted with 105 mm focal length macro lenses, and a LaVision PTUx timing unit. This equipment was located in the test room, with the air needed for camera and laser cooling supplied through dedicated ventilation ducting connected to a nearby sealed doorway, ensuring no air exchange with the test environment. Control and data acquisition were performed from the exterior measurement station using LaVision's DaVis 10.0 software. The ventilator limit switch signals were passed into the PIV timing unit, allowing for measurement synchronization with the breathing cycle.

Measurements involving the manikin were performed in two locations, both depicted in Fig. 2(a), with the measurement planes located at the mid-span of the test model (within the *x*-*y* plane). The first measurement field of view (FOV) measured 350×650 mm, covering the area of exhaled breath for both masked and unmasked cases, and was imaged using two cameras, each at a magnification factor of 0.04. Here, flow visualization and PIV images were acquired at 15 Hz, with the latter requiring aerosol seeding of both the breathing stream and ambient environment. Double-frame PIV images were acquired using frame separation times between 15 and 20 ms, resulting in particle displacement below approximately 20 pixel. The particle images were then processed in DaVis 10 using sliding background subtraction and intensity normalization, followed by an iterative, multi-pass cross correlation algorithm with a final window size of 32×32 pixel (50% overlap) to determine local flow velocities at a spatial resolution of 2.98 mm.

At the second measurement location, a single camera was used to image a 107×90 mm FOV centered at the height of the exhalation point (1.1 m from the floor) but at a 2 m distance [Fig. 2(a)]. Here, single images were acquired at a rate of 0.25 Hz for up to 10 h in order to track the dispersion of exhaled aerosols from the test subject. In order to minimize the gradients of light intensity within the image, the laser sheet was expanded substantially larger than the dimensions of the field of view (approximately 200% more), such that the core region of the laser beam covered the entire field of view. The directivity of dispersion was investigated by rotating the manikin about the *y*-axis while keeping the measurement location fixed, with orientation angles of 0°, 90°, and 180° considered. For each case, imaged particles (2–3 pixel in the imaging plane) were detected and counted using a particle detection algorithm in DaVis 10.1 software, providing the measure of local particle concentration based on the average over the local measurement volume. The particle detection algorithm measures particle counts by scanning the image for peaks in local intensities after the image is pre-processed using a sliding minimum subtraction and low-pass Gaussian filter to enhance the individual intensity peaks. A threshold for the background noise is employed and kept constant between all the cases for consistency.

A total of seven PPE configurations were considered, with the manikin fitted with (i) no mask, (ii) an unvalved KN95 mask, (iii) a typical three-ply blue pseudo-surgical mask, (iv) a three-ply cotton cloth non-medical mask, (v) a 3M R95 particulate respirator (equivalent to N95 for human borne aerosols), (vi) an unvalved KN95 mask with 3 mm gaps around cheeks and nose, and (vii) a KN95 mask with a single one-way valve on the left side. The parameters presented in Table I were kept constant across all cases. To adjust for a higher average rigidity of the manikin face and have repeatable mask fits, straps typically worn around the ears were tightened by anchoring them to a single peg located inline with the top of the ears and at the center of the back of the head. Note that this was not the case for the R95 respirator, which has straps that circumnavigate the head and neck. Tests with the KN95 mask with artificial gaps [case (vi)] were performed by first ensuring the same baseline fit as that of the unvalved KN95 mask [case (ii)], with the gaps created by 3 mm thick pieces of vinyl foam placed on the cheeks and cheekbones of the manikin, which produced consistent leakage sites similar to the approach employed by Weber *et al.*^52^ The length of the foam pieces was minimized to reduce blockage while ensuring consistent gap dimensions between multiple runs.

Tests characterizing breath particle concentration, breath volume, and ideal mask filtration efficiency were also performed, with the setups used presented in Figs. 2(b) and 2(c). These tests utilized the same breathing apparatus, breathing parameters, and imaging setup as the dispersion tests, with the outlet of the ventilator fed to a 25 mm inner diameter rigid pipe. For characterization of the breath particle concentration, the outlet of the pipe was fed into an acrylic box (dimensions 0.6×0.6×0.9 m) which was sealed off after a single breath and images were acquired at 1 Hz for 0.5 h. A uniform particle distribution was reached after approximately 15 min, after which the number of particles was measured. From this, and the FOV area and laser sheet thickness (2.0 mm), the total number of particles contained in the volume and therefore injected by the breath was estimated, resulting in the value reported in Table I. The provided uncertainty range is based on the variance found across ten runs of repeatability.

For breath volume, ideal mask filtration efficiency, and mask pressure drop characterizations, the outlet of the pipe was exhausted to open air and the measurement field of view was moved to the exit of the pipe, as shown in Fig. 2(c). Mask material was sealed around the pipe outlet using a 3 mm thick o-ring and hose clamp. PIV double-frame measurements with a frame separation times of 666 μs were acquired at 15 Hz for the duration of the exhalation over 50 cycles. Image processing was performed in DaVis 10 using sliding background subtraction and intensity normalization, followed by vector calculation using iterative, multi-pass cross correlation with a final window size of 24×24 pixel (75% overlap), yielding a spatial resolution of 0.25 mm. The resulting velocity field data were integrated to give phase average volumetric flow rate, yielding the total breath volume reported in Table I. A pressure tap flush with the interior of the pipe wall was installed two diameters upstream of the pipe exit and was connected to a Setra pressure transducer (Model 264), providing static pressure measurements relative to the local atmospheric pressure. For ideal mask filtration efficiency, the total number of particles exhausted over an exhalation cycle was counted. The result was then compared to the case with no mask, with 50 breath cycles used to establish a confidence interval.

## III. RESULTS AND DISCUSSION

This section first discusses the ideal filtration characteristics of various masks used in the present study. Thereafter, the near-field flow visualization and velocity measurements around the face of the test subject are discussed. Finally, the results corresponding to particle dispersion in the test room are presented along with the supporting model results.

### A. Baseline mask characteristics

**FIG. 3.**
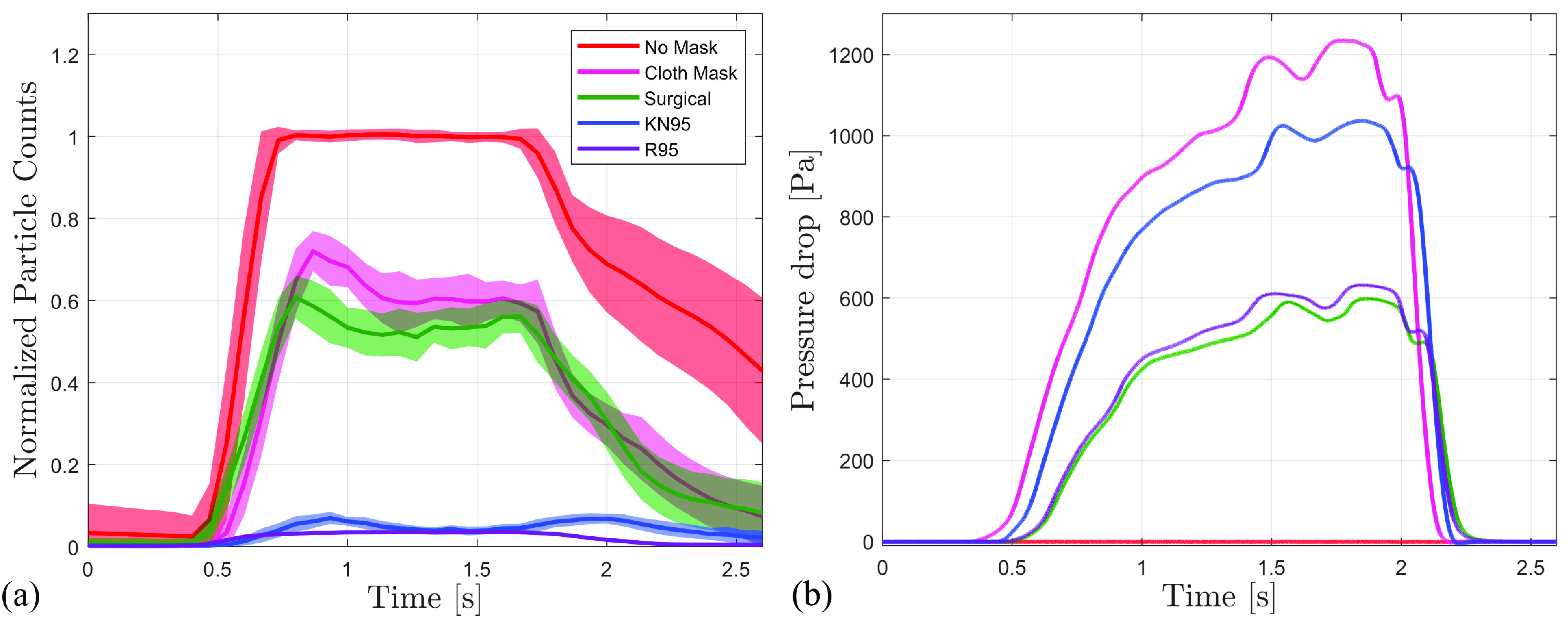
(a) Particle counts after aerosol particles are passed through various masks and normalized by the particle counts at the plateau in the no-mask case, and (b) associated pressure drop across the mask at a flow rate of $0.4\;L\;s^{-1}$. Results are from the setup shown in Fig. 2(c) and are averaged over 50 exhalation cycles. Shaded areas correspond to ±1 standard deviation. The standard deviations for pressure drop measurements are negligible, and thus, not visible.

Significant variability in essential mask characteristics has been reported in previous studies, which tends to be more significant for non-certified mask types. Thus, baseline parameters for each mask type considered in the present study have been established experimentally and ensured to be consistent for the same mask types tested here. The baseline ideal filtration characteristics of the studied masks are established for the breathing parameters (Table I) and aerosol employed in the study. An estimate of ideal filtration efficiency, and the associated pressure drop across the mask, is established through tests where the mask is sealed at the point of exhaust [Fig. 2(c), as described in Sec. II], thereby removing the dependency on mask fit to the test model. The results are presented in Fig. 3(a), showing the particle concentration during exhalation, with results averaged over 50 cycles and normalized by the peak concentrations reached in the unfiltered case (no mask).

In Fig. 3(a), for the no-mask case, exhalation begins at approximately 0.5 s, with particle counts downstream of the outlet increasing rapidly after the initiation of the exhalation, followed by an extended period of stabilization at the peak value, and a subsequent decrease in the particle concentration toward the end of the exhalation cycle. A similar trend is observed for the tested mask cases, with lower plateau values reached as a result of filtration. The ideal filtration efficiency for a mask is estimated by computing the change in the average particle concentration within the time interval of 1 and 1.5 s, i.e., the plateau value, relative to the no-mask case, with obtained results summarized in Table II. The efficiency of the KN95 and R95 masks is the highest at approximately 95% and 96%, respectively, which agrees well with the rated efficiencies for these masks in the absence of leakage. Such high efficiencies are attributed to the electrostatic filters embedded in these masks, which have been shown to effectively filter both charged and neutrally charged particles.^53,54^ Filtration efficiencies for the blue surgical mask and cloth masks are significantly lower at 47% and 40%, respectively, meaning that more than half of the aerosol particles pass through these masks. The present results are in reasonable agreement with Jung *et al.,*^55^ who compared filtration efficiencies of a number of medical and non-medical masks. Note that a relatively wide variation of filtration efficiencies has been reported for these types of PPE in previous studies,^56–58^ largely attributed to the lack of stringent filtration performance standards.

**TABLE II.** Filtration characteristics of various masks at an integrated flow rate of 0.4 L s^−1^. ΔP and *P_dyn_* indicate the peak pressure drop and the peak dynamic pressure, respectively, obtained at the peak flow rate ( Q= 0.61 L s^−1^). The 95% confidence intervals on the mean filtration efficiencies and peak pressure drop are within ±1.5% and ±0.25%, respectively, for all the cases.

Mask material	Filtration efficiency (%)	ΔP (Pa)	ΔP/*P_dyn_*	ΔP/*Q* ( $\text{Pa}\;{s/m}^{3}\times 10^{-5}$)
Cloth	40	1196	1356	19.67
Surgical	47	573	650	9.42
KN95	95	1014	1150	16.68
R95	96	606	687	9.97

Pressure drop across a mask and the corresponding flow resistance coefficient ( ΔP/Q, where *Q* is the peak flow rate) are important considerations since both provide measures of mask breathability and, consequently, comfort when worn by an individual, with a lower pressure drop and resistance coefficient indicating higher comfort. The results in Fig. 3(b), along with the parameters summarized in Table II, show that the KN95 and cloth masks have the highest pressure drops and resistance coefficients, indicating relatively poor breathability. In comparison, the pressure drop across the R95 mask is approximately 40% lower than that of the KN95 mask, which is significant given a similar level of filtration efficiency. Pressure drop across the surgical mask is comparable to that of the R95, indicating a similar level of breathability and comfort; however, this comes at the cost of significantly reduced filtration efficiency. It should be noted that substantial variability in measured pressure drop can occur even for the same mask types from different manufacturers;^26,56,57,59,60^ however, the trends observed in the present measurements fall within the range of values reported previously. Therefore, these results can serve as a qualitative guide toward the balance between ideal filtration performance and breathability for common face masks.

### B. Exhalation flow characterization

**FIG. 4.**
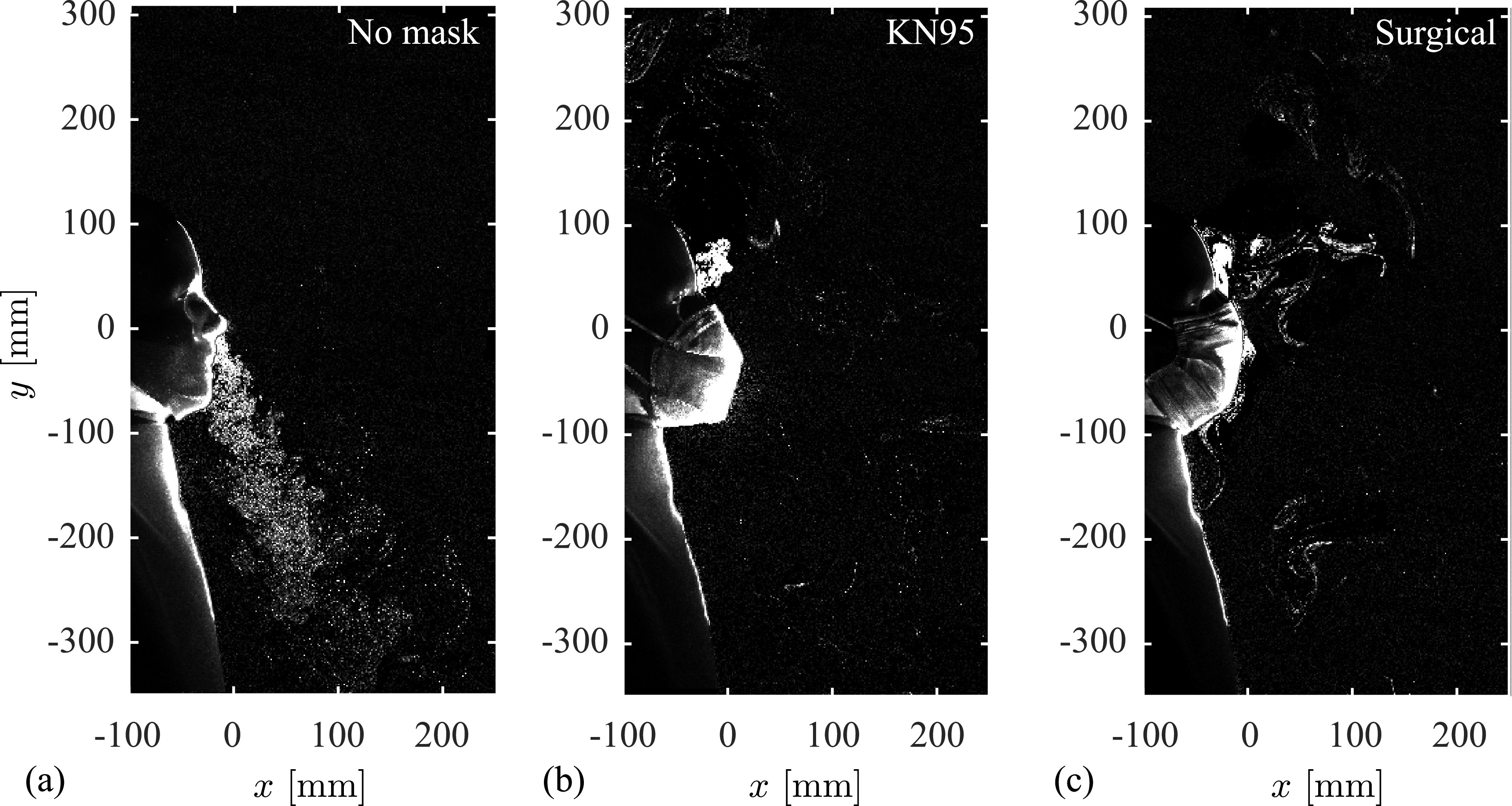
Instantaneous flow visualizations at 180° within the breathing cycle for the (a) no mask, (b) KN95, and (c) surgical mask cases. Multimedia views: https://doi.org/10.1063/5.0057100.1
10.1063/5.0057100.1; https://doi.org/10.1063/5.0057100.2
10.1063/5.0057100.2; https://doi.org/10.1063/5.0057100.3
10.1063/5.0057100.3

With baseline filtration characteristics of the masks established, their effect on the evolution of exhaled breath through the nose of the test model is now considered in the vicinity of the face using particle flow visualization and velocimetry techniques. Results for the KN95 and surgical mask are seen to qualitatively represent a high-efficiency mask and common cloth/non-medical masks, respectively. Thus, these two configurations are used here as representative face mask groups, and the results are contrasted with the no-mask case. Figure 4 illustrates nasal exhalation through an instantaneous flow visualization image at the vertical mid-plane of the face and at a phase angle of 180° within the breathing cycle (exhalation begins at 0°). Multimedia views included for each case depict the flow development over a few breathing cycles. The exhaled flow in the case of no mask [Fig. 4(a) (Multimedia view)] is typical of a transient turbulent jet, with the expelled aerosols directed downwards and the jet front reaching a distance from the nose of approximately 300 mm within approximately 1 s. The turbulent nature of the jet is apparent, with small scale eddies, visualized by particle clouds, present throughout the jet core, with the darker patches around the jet perimeter showing fluid entrained into the jet by turbulent mixing. In fitting the manikin with a mask, both the KN95 and surgical masks [Figs. 4(b) (Multimedia view) and 4(c) (Multimedia view), respectively] are successful in arresting nearly all forward momentum of the exhaled jet. As noted across the literature,^61–63^ this is the primary protective mechanism of a mask for direct exposure to aerosols as it serves to reduce and redirect the forward momentum of the exhaled breath, which, as will be shown in Sec. III C, has a significant effect on the dispersion of exhaled aerosols away from the subject over time.

It is important to note that, while masks [Figs. 4(b) (Multimedia view) and 4(c) (Multimedia view)] decrease the forward momentum of the respiratory jet, a significant fraction of aerosol escapes the masks, particularly at the bridge of the nose. Further, aerosols can also be seen in front of the surgical mask due to the lower material filtration efficiency (Table II). These leakages are more readily apparent in the multimedia views. Recent studies employing similar visualization techniques for other types of expiratory events, such as sneezing, coughing, laughing, and speaking,^32,61,64^ show similar leakage through surgical and common cloth masks. In those studies, higher pressure differences were imposed and therefore particles passing through the mask may have been expected, while the present results highlight that the pressure difference created by normal breathing is sufficient to cause aerosols to pass through the fabric of a surgical mask. In contrast, such leakage is negligible in the KN95 case [Fig. 4(b) (Multimedia view)], which is representative of high quality, certified masks.

As previously noted, a significant quantity of aerosol escapes at the bridge of the nose in Figs. 4(b) (Multimedia view) and 4(c) (Multimedia view), which highlights the importance of the fit of the mask to the face. Here, the fit of each mask is typical of appropriate usage, with the straps tightened (as outlined in Sec. II) and the built-in wire shaped to the bridge of nose. Nonetheless, aerosols escape at the perimeter of the mask due to inevitable imperfections in the mask fit, with the most significant quantity of particles escaping at the bridge of the nose. Other leakage sites include the interface of the mask edges with the cheeks and lower jaw [not captured in Figs. 4(b) (Multimedia view) and 4(c) (Multimedia view) due to laser sheet positioning]; however, these results and other supplementary measurements (not shown for brevity) confirm that leakage at the bridge of the nose far exceeds all other leakage points. At the bridge of the nose, the particle clouds that escape the masks are relatively dense in comparison to the exhaled jet in the no-mask case, which is attributed to the significant redirection of momentum needed to force particles out at the top of the mask, resulting in much lower exit velocities and hence reduced turbulent diffusion. The observed particle concentrations just outside the mask qualitatively agree with the results of Sickbert-Bennett *et al.*^65,66^ who obtained fitted filtration efficiency (FFE) estimates of more than 95% for inhalation with N95 type masks. However, their FFE estimates are based on the particle concentration entering the mask from the ambient air and are not directly indicative of the mask efficiency when considering the exhalation of aerosols. The results in Fig. 4 illustrate that a notable amount of particles leak out at the perimeter of all masks, which is expected to result in notably lower effective filtration efficiency, compared to ideal filtration efficiency, when exhalation is considered.

**FIG. 5.**
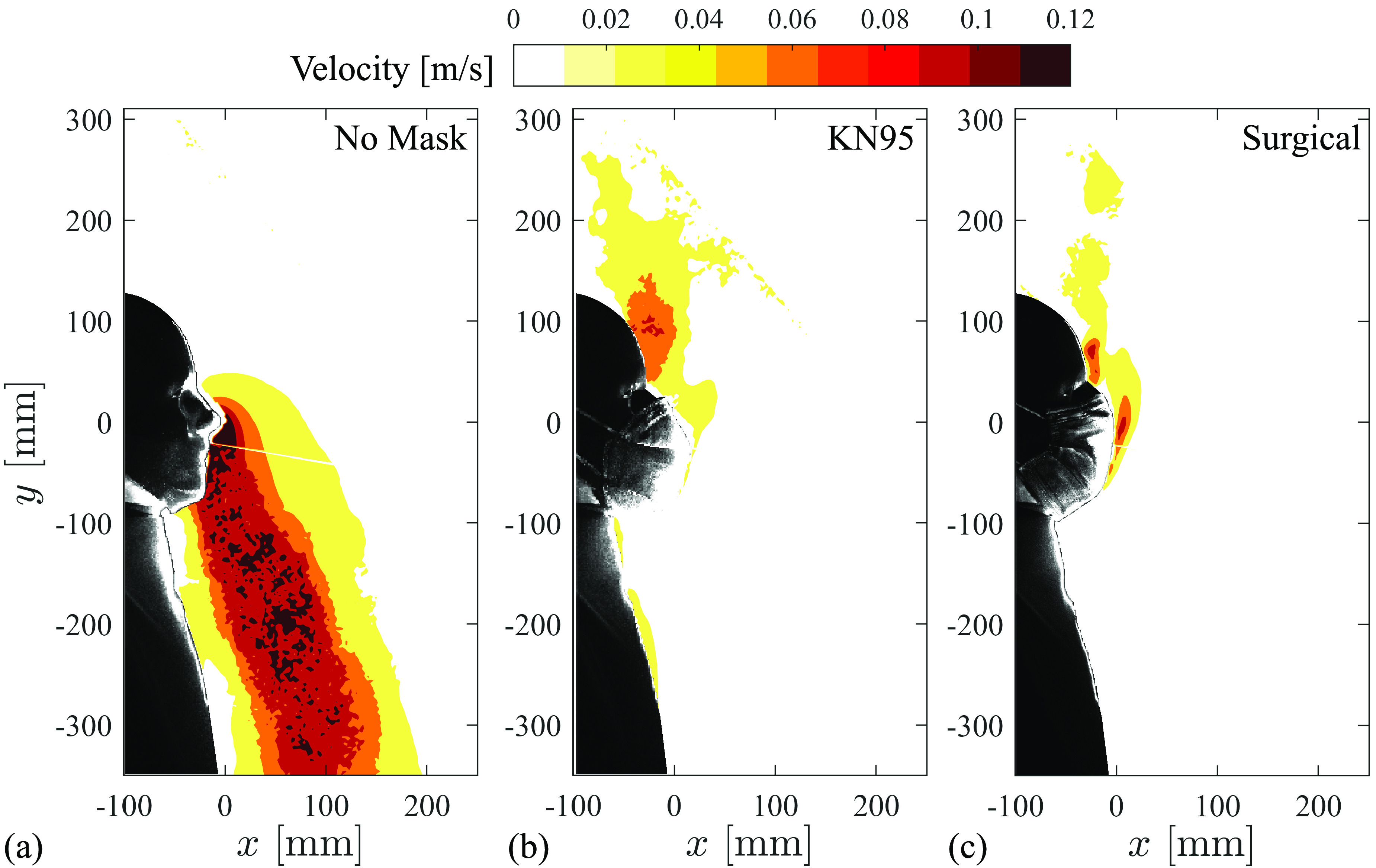
Phase-averaged velocity fields at 180° within the breathing cycle for the (a) no mask, (b) KN95, and (c) surgical mask cases. Multimedia views: https://doi.org/10.1063/5.0057100.4
10.1063/5.0057100.4; https://doi.org/10.1063/5.0057100.5
10.1063/5.0057100.5; https://doi.org/10.1063/5.0057100.6
10.1063/5.0057100.6

Figure 5 presents phase-averaged velocity fields, again at a phase angle of 180° within the breathing cycle, matching Fig. 4. Multimedia views are also provided for each case, showing phase-averaged velocity field development over the full exhalation cycle. Note that these measurements were performed at the mid-plane of the manikin face, not at the center of a given nostril. For the case with no mask [Fig. 5(a) (Multimedia view)], typical turbulent jet characteristics are noted, with jet propagation and spreading rate typical of accelerating jet flows.^67^ Within the measurement plane, peak velocities range from 0.10 to 0.12 ms^−1^ in the core of the jet, which is within the range of velocities investigated in previous studies for normal breathing.^53,56,68–71^ The results confirm that the forward momentum is decreased dramatically when the subject is fitted with a mask [Figs. 5(b) (Multimedia view) and 5(c) (Multimedia view)], as was seen in the flow visualizations (Fig. 4). For these cases, the expelled flow is directed primarily upward and backward by the mask and remains attached to the forehead due to the Coanda effect, with peak velocities reduced to less than 0.10 ms^−1^. For the surgical mask, the flow that penetrates through the front of the mask is of relatively low forward momentum and, consequently, much lower penetration depth, as seen in Fig. 4(c) (Multimedia view). Together, the flow visualization and PIV results (Figs. 4 and 5, respectively) highlight important safety aspects when considering aerosols dispersed by an individual's breathing. When not fitted with a mask, exhalation from the nose produces a relatively strong turbulent jet containing well mixed particles that will disperse relatively quickly away from the subject. While in the case of equipping a mask, the jet momentum is significantly reduced and redirected, leading to leakages of aerosols at any point where the mask does not maintain a tight seal to the face. Based on the results obtained here, the leakages are most significant at the bridge of the nose, leading to dense aerosol clouds exiting near and remaining close to the fore and top of the head.

### C. Aerosol dispersion in an indoor environment

Noting the significance of both the ideal filtration characteristics (Sec. III A) and fit of a mask (Sec. III B), it is apparent that both effects must be taken into account in order to provide an accurate measure of the effectiveness of a mask in reducing the dispersion of an aerosol exhaled by an individual. This is investigated through the measurement of aerosol dispersion from the test model in a vacant indoor space over a period of 10 h, with the particle concentration measured at a 2 m distance from the subject [Fig. 2(a)], aligned with the widely accepted social distancing recommendations.

In an enclosed space with negligible convective effects, such as the room in which the tests are conducted, the concentration of dispersed aerosols away from the source is governed by the unsteady diffusion equation

$$\frac{dc}{dt}=\nabla \cdot (K\nabla c)+R-\lambda c,$$ (1)

where *c* is the concentration of aerosol particles (particles m^−3^), *t* is time, *K* is the diffusion coefficient (m^2^ s^−1^), *R* is the particle injection rate (particles m^−3^ s^−1^), and the sink term containing the decay rate *λ* (s^−1^) which takes into account particle decay.^35^

While Eq. (1) has been used for modeling in a number of previous studies,^17,32,35,72^ the model outcomes are predicated on appropriate estimation of the injection rate, decay, and diffusion terms, with the commonly employed coarse estimates only providing qualitative understanding of the spatial and temporal evolution of particle concentration for various room and source configurations. In practice, it is extremely challenging to obtain reasonable estimates for these values,^32^ while computational results remain extremely sensitive to these parameters.

A significant simplification to Eq. (1) is commonly employed by assuming instantaneous distribution of produced aerosols in the room as in the following equation:

$$\frac{dc}{dt}=R-\lambda c.$$ (2)

The solution to Eq. (2), subject to the initial condition $c^{*}(t=0)=0$, is given by

$$c^{*}(t)=\frac{R^{*}}{\lambda^{*}}(1-e^{-\lambda^{*}t}).$$ (3)

For the purposes of practical data assimilation considered in the present study, the underlying simplification absorbs the effect of diffusion into the sink and source terms. This makes the solution dependent on the spatial location, and the relevant parameters are marked with an asterisk ( $c^{*},\;R^{*},\;\lambda^{*}$). Equation (3) models the temporal evolution of concentration in a typical first-order fashion with a saturation concentration of $c_{\text{sat}}^{*}=R^{*}/\lambda^{*}$. Although previous studies have noted significant deviations of diffusion-based computational results from the well-mixed model,^72–74^ the simplified model will be shown to fit well with the experimental data and thus provides a suitable comparison basis for saturation conditions. The latter allows for relative source strength comparisons between different test cases, which is of particular importance for the evaluation of the apparent mask filtration efficiency.

**FIG. 6.**
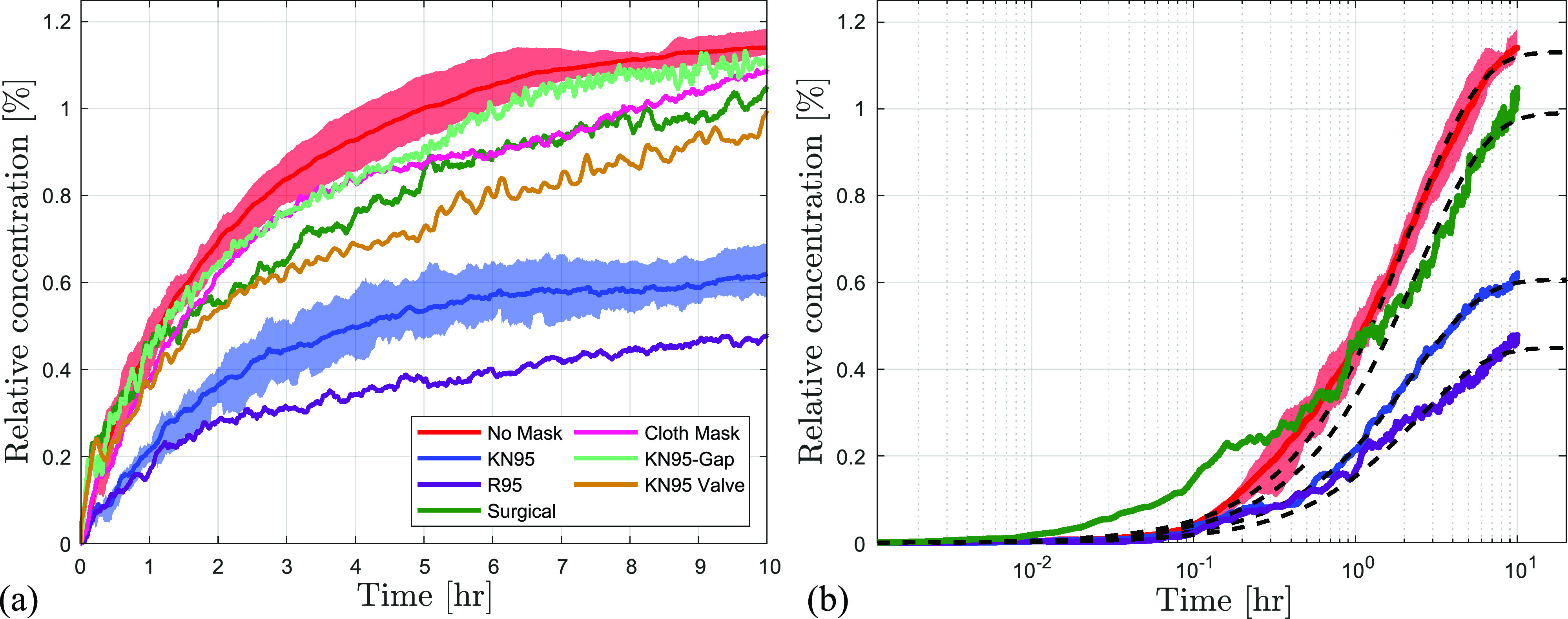
Effect of various masks on the dispersion of aerosols measured at 2 m in front of the manikin. (a) Results are smoothed using a 10 min moving average. (b) Moving average particle concentration on semi-logarithmic scale for selected cases with a black dashed line showing model fits. Shaded areas show variability within repeated runs.

Experimental results from the aerosol dispersion tests are presented in Fig. 6, with results normalized by the average particle concentration of a single breath (Table I) and smoothed using a 10 min moving average. For clarity, the variability between repeated measurements is illustrated by the shaded regions only in the no-mask and KN95 cases, which are representative of the typical variability observed in all the tested cases. In Fig. 6(b), the results are also plotted on a logarithmic scale and are fitted based on the typical first-order behavior described by Eq. (3). The obtained least squares fit parameters are presented in Table III, with the corresponding confidence intervals determined based on repeated tests.

**TABLE III.** Apparent filtration characteristics of various masks based on particle dispersion tests over 10 h. $R^{*}$ ((%/h) of the breath particle concentration) and $\lambda^{*}$ (h^–1^) are fit parameters estimated using a multi-variable least squares fit of Eq. (3) to the experimental data in Fig. 6. Values for the parameters are shown with a 95% confidence interval based on the t-statistic. Confidence interval on $\eta_{\text{AFE}}$ incorporates the variation in the no-mask case.

Material	$R^{*}$ ( %h)	$\lambda^{*}$ (h^–1^)	$c_{\text{sat}}^{*}=R^{*}/\lambda^{*}$ (%)	$\eta_{\text{AFE}}$ (%)
No mask	0.53 ± 0.11	0.46 ± 0.11	1.13 ± 0.057	⋯
Cloth	0.45 ± 0.27	0.44 ± 0.31	1.02 ± 0.11	9.8 ± 9.7
Surgical	0.41 ± 0.36	0.41 ± 0.39	0.99 ± 0.11	12.4 ± 9.7
KN95	0.27 ± 0.10	0.45 ± 0.12	0.61 ± 0.095	46.3 ± 9.4
R95	0.19 ± 0.09	0.42 ± 0.11	0.45 ± 0.09	60.2 ± 9.0
KN95-gap	0.46 ± 0.16	0.42 ± 0.21	1.09 ± 0.09	3.4 ± 8.9
KN95-valve	0.37 ± 0.12	0.41 ± 0.14	0.90 ± 0.09	20.3 ± 8.9

It can be seen that the simplified model captures the essential concentration trends well. The average relative concentration in the no-mask case is seen to asymptotically tend to the local steady state value of 1.13% of the breath particle concentration after a period of 10 h. Upon fitting various masks to the manikin, the relative concentrations are lowered in comparison to the no-mask case, indicating a reduction in the source strength due to filtration. The same is also captured in the reduction of the relative particle injection rate. However, the relative changes in the injection rate are significantly lower than those expected purely based on the ideal filtration efficiency of the mask material (Table II), which is attributed to the substantial aerosol leakage seen in Fig. 4.

Given close adherence of the experimental data to Eq. (3), the estimated saturated, i.e., steady state, concentration levels can be used to deduce the apparent filtration efficiency of the masks,

$$\eta_{\text{AFE}}=100\times \left(\frac{c_{{\text{sat}}_{\text{NoMask}}}^{*}-c_{\text{sat}}^{*}}{c_{{\text{sat}}_{\text{NoMask}}}^{*}}\right).$$ (4)

The resulting estimates for the apparent filtration efficiency ( $\eta_{\text{AFE}}$) are reported in Table III, which confirms that $\eta_{\text{AFE}}$ for all the masks is significantly lower than the filtration efficiencies for their respective materials presented in Table II. The R95 mask has the highest $\eta_{\text{AFE}}$ of 60.2%, which is attributed to the tighter fit of the mask obtained by the overhead straps, a relatively stiff fabric, and the built-in soft sealing layer at the nose bridge of the mask. For KN95 mask, the gaps along the cheeks and the nose bridge are found to be comparatively larger, which leads to a lower $\eta_{\text{AFE}}$ despite a similar filtration efficiency of the material. The cloth and surgical masks perform relatively poorly with efficiencies of only 9.8% and 12.4%, respectively, due to both low material filtration efficiency and significantly higher amounts of leakages around the cheeks and bridge of the nose. Further, due to the higher flexibility of the cloth and surgical mask material, they easily deform during exhalation, causing an increase in the size of the preexisting gaps, allowing more aerosols to escape.

In order to further evaluate the effect of leakage through the gaps around the cheeks and the nose, a separate case with the KN95 mask was considered with 3 mm gaps created artificially, as described in Sec. II. The 3 mm gaps are representative of the typical gaps observed for the surgical and cloth masks and provide a “loose-fitting” KN95 case. Results for the KN95-gap case in Fig. 6 and Table III show a significant reduction in the filtration efficiency compared to the baseline KN95 mask, with $\eta_{\text{AFE}}$ decreasing from 46.3% to a paltry 3.4%. This offers a holistic perspective on the implications of loose fitting masks and aerosol build-up, in contrast with the results of Sickbert-Bennet *et al.*^65^ whose single-point measurement directly behind the mask shows an efficiency (FFE) of more than 90% with a sub-optimally fit N95 mask. An additional point of comparison is provided in the present study by an appropriately fitted KN95 mask equipped with a one-way valve, which has an apparent efficiency of approximately 20%. This illustrates that controlled discharge through a valve on a high-efficiency mask may lead to a better overall exhale filtration compared to either a lower-grade mask (cloth or surgical) or a loosely fitted high-efficiency mask.

**FIG. 7.**
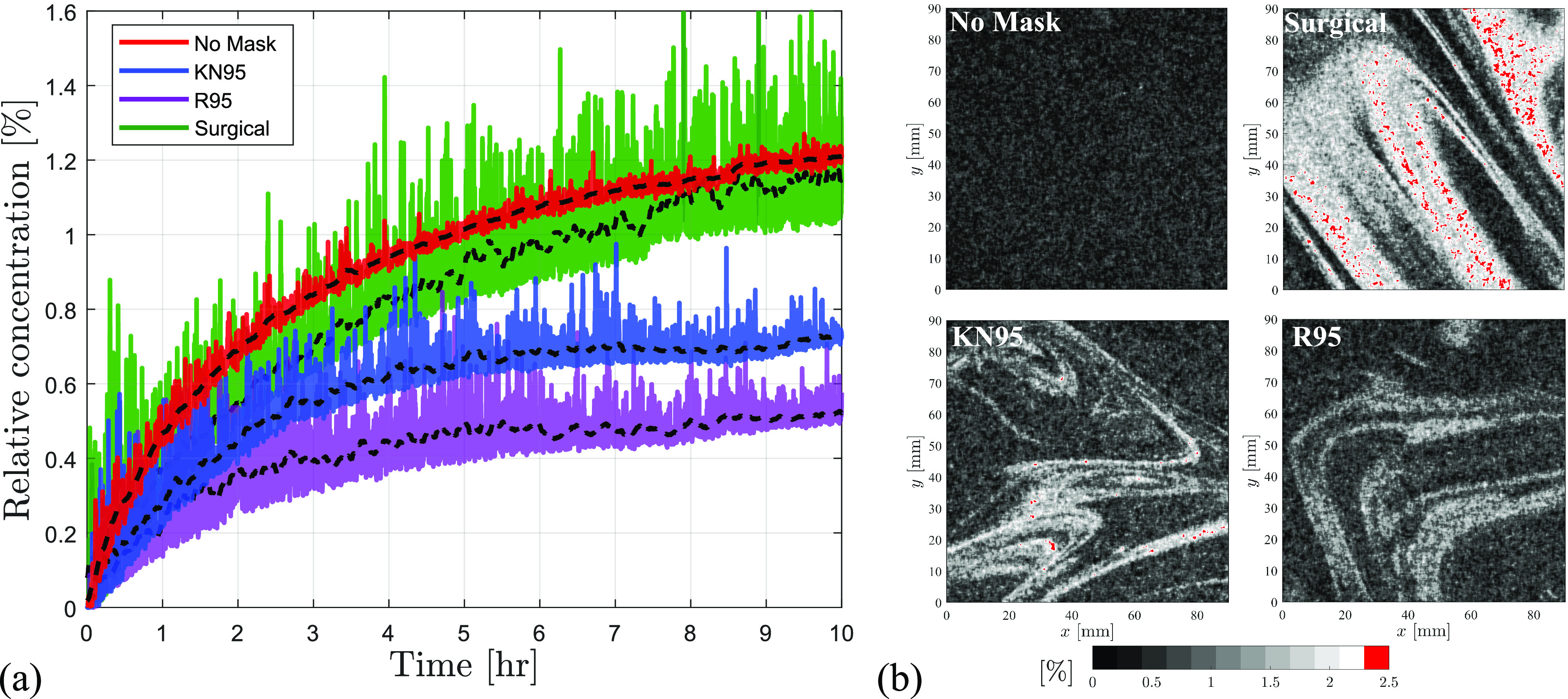
(a) Raw particle concentration for representative test cases considered in Fig. 6 with moving average shown with black dashed lines. (b) Instantaneous relative particle concentration fields for the selected cases. Relative concentrations are represented as a fraction of the breath particle concentration.

An important aspect of mask usage that is not apparent in Fig. 6 due to temporal smoothing and averaging over repeated runs is illustrated in Fig. 7, which presents raw particle concentrations for a selected subset of test cases. The instantaneous particle concentrations measured within the field of view in Fig. 7(a) show large temporal variations in local concentrations when masks are used, which consistently exceed those seen for the no-mask case. The instantaneous magnitudes of particle concentrations reach up to 1.6% of the single breath concentration in the case of blue surgical mask, roughly 40% above the saturation concentration reached in the no-mask case. These maximum excursions in the cases of the KN95 and R95 masks are lower; however, the instantaneous spikes in concentration surpass the average no-mask concentration in the first hour of the test. These excursions in the local particle concentrations are attributed to the presence of dense particle clouds that frequently pass through the field of view, as illustrated in Fig. 7(b). The figure shows representative concentration maps of the particle clouds in the blue surgical and the KN95 mask cases. Peak concentrations reach up to 3% of the particle breath concentrations in the blue surgical mask case, which are localized within the core regions of the clouds and indicate a much higher threat than that perceived based on the averaged results in Fig. 6. Although these particle clouds were present in every tested case with a mask, their frequency and sizes decreased for masks with better fits and higher apparent filtration efficiencies ( $\eta_{\text{AFE}}$), as illustrated by representative realizations for KN95 and R95 masks in Fig. 7(b). The implication for disease mitigation is a significant temporal variability in the exposure risk associated with masks in an unventilated indoor environment. Recent studies^72,73^ have noted similar concentration excursions attributed to the local flows, exceeding the predictions based on the well-mixed and diffusion based models.

**FIG. 8.**
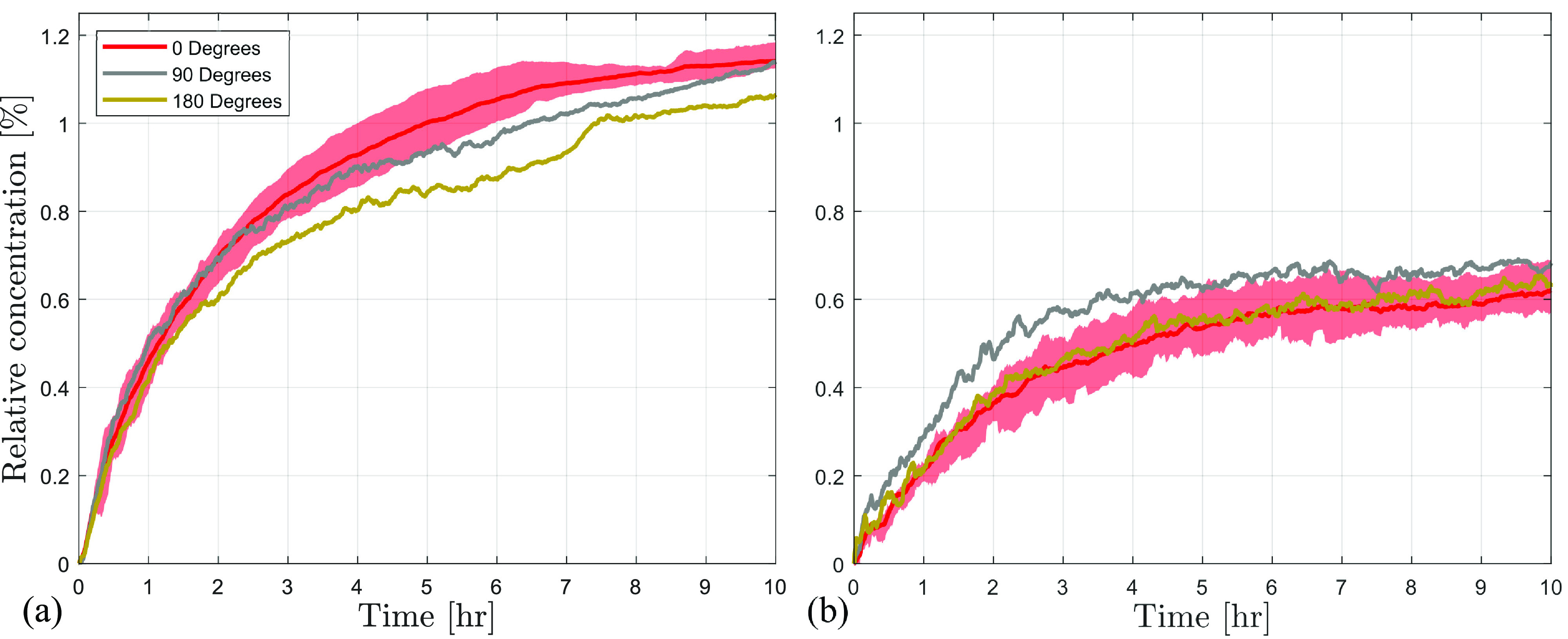
Dispersion characteristics of the aerosols measured at a radius of 2 m from the manikin for (a) no mask and (b) KN95 mask.

It is of practical interest to investigate the directivity of the exhaled particles for social distancing purposes in indoor environments with poor ventilation. Directivity of the particle dispersion at the 2 m distance from the source was investigated in the no-mask and KN95 cases, and the results are presented in Fig. 8. The results for the no-mask case in Fig. 8(a) show that the average concentrations reached at 90° and 180° decrease in comparison to those at 0°, but the effect of the orientation is less than 10%. In the case of KN95 mask [Fig. 8(b)], the particle concentrations at the non-zero orientations are only slightly higher than those at 0°. While the general trend highlighted by these results is in accordance with the expectations based on the flow visualization results (Fig. 4), the differences with orientation are relatively minor which indicates that the anticipated effect of directivity due to advection is primarily confined to the near vicinity of the source. In the absence of ventilation effects, turbulent diffusion appears to largely equalize the concentration along the circumferential direction at and beyond the 2 m radial distance surrounding the source. This is in accordance with the typical deposition mechanisms observed in the case of suspended particles.^75^ However, the observed effect may be limited to relatively large room sizes, such as those used in the current experiment, where the advection effects near the source become negligible well inside the boundaries of the room. The current results are in qualitative agreement with the results from diffusion based models^35^ in a poor ventilation scenario.

**FIG. 9.**
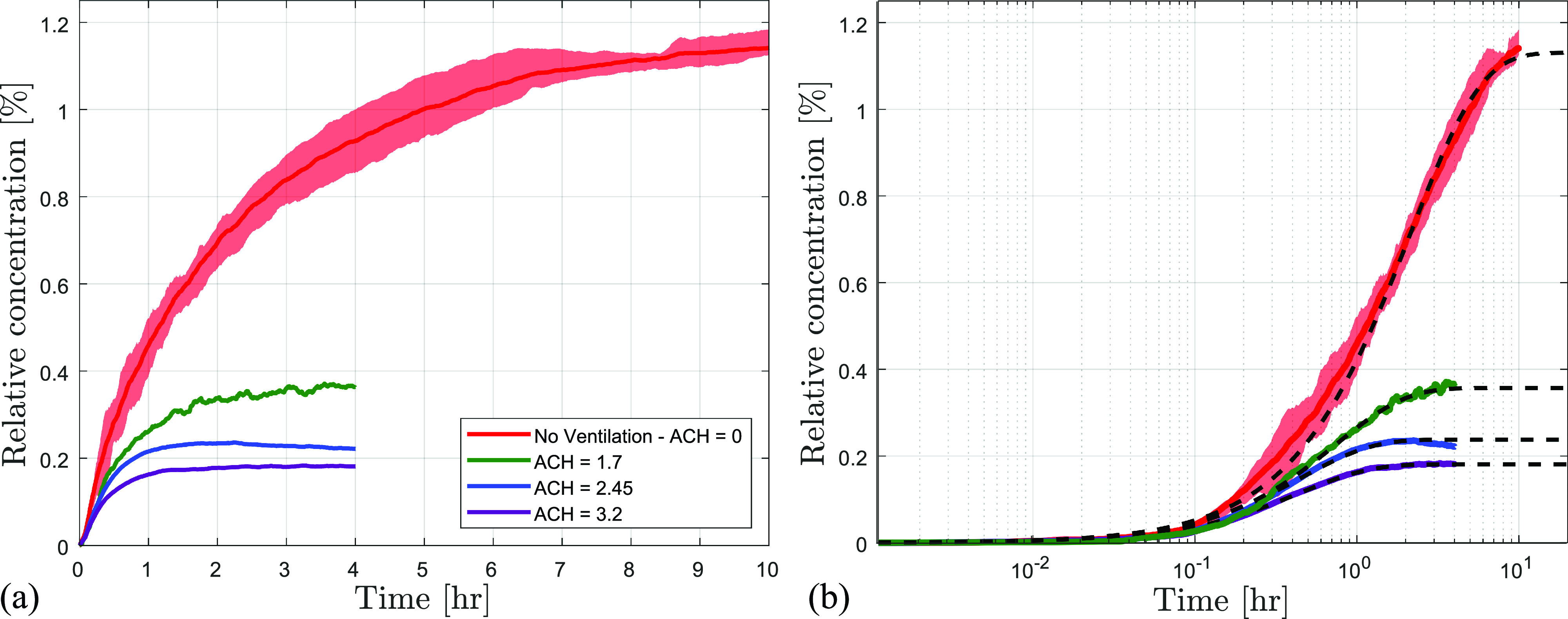
Effect of ventilation on the dispersion characteristics of the aerosols measured at 2 m in front of the manikin without a mask on a (a) linear, and (b) logarithmic scale in time. Black dashed line indicates model fits to the moving average data.

Finally, the effect of room ventilation and/or air cleaning is investigated on the aerosol dispersion 2 m in front of the manikin. Measurements are conducted at three different settings of a mobile air purifier installed in the corner of the room (left top corner in Fig. 1). Due to a high efficiency particle air (HEPA) filtration ( >99% efficiency), the unit allows a controlled modeling of ventilation settings, with effective air-change rates (ACH) of 1.7, 2.45, and $3.2\;h^{-1}$ considered in the present investigation. The results presented in Figs. 9(a) and 9(b) show a notable reduction in local concentration in front of the manikin even with relatively low effective air-change rates, as also noted by previous studies^35,73^) The measured concentrations are seen to decrease with increasing ACH, and the steady-state ( $c_{\text{sat}}^{*}$) is achieved within less than 4 h in all the air-cleaning cases. The results are fitted to the simplified model [Eq. (3)] in Fig. 9(b), and the fits are seen to approximate the data well. The corresponding fit parameters are summarized in Table IV. As expected, the increase in ACH results in a notable increase in the decay rate ( $\lambda^{*}$), which is reflected in the earlier saturation of the local concentration. This is also in accordance with the increased diffusion coefficient in mixing ventilation scenarios as shown by Foat *et al.*^72^ and Cheng *et al.*^76^ at comparable ACH. The steady-state values are used to estimate an apparent filtration efficiency ( $\eta_{\text{AFE}}$) of the system in order to draw meaningful comparisons with the results from the mask cases in an unventilated scenario. In this case, the apparent filtration efficiency ( $\eta_{\text{AFE}}$) is obtained by the relative change in the steady-state concentration ( $c_{\text{sat}}^{*}$) between the ventilated and unventilated cases. The results in Table IV show that the steady-state concentrations are decreased in the range 69%–84% for the considered cases and correspond to a much higher $\eta_{\text{AFE}}$ compared with the best performing mask in an unventilated scenario (Table III). However, this also suggests that relatively low ventilation rates (ACH $<\;3.2\;h^{-1}$) may not be sufficient to reduce exposure to within acceptable levels at the typical social distancing guideline of 2 m, which supports the findings from previous studies.^73,77^

**TABLE IV.** Apparent filtration efficiencies for various air-change rates (ACH) based on particle dispersion tests with no-mask. $R^{*}$ ((%/h) of the breath particle concentration) and $\lambda^{*}$ (h^–1^) are fit parameters estimated using a multi-variable least squares fit of Eq. (3) to the experimental data in Fig. 9.

ACH	$R^{*}$ ( %/h)	$\lambda^{*}$ (h^–1^)	$c_{\text{sat}}^{*}=R^{*}/\lambda^{*}$ (%)	$\eta_{\text{AFE}}$ (%)
0	0.53	0.46	1.13	⋯
1.7	0.48	1.36	0.35	69
2.45	0.52	2.19	0.24	79
3.2	0.41	2.27	0.18	84

## IV. CONCLUSIONS

The present study experimentally investigates the dispersion and accumulation of aerosol particles in indoor environments in the context of the guidelines proposed by national health agencies to control the transmission of COVID-19. Experiments were conducted in a controlled laboratory environment with a test manikin in a seated position mimicking relaxed exhalation through the nose, typical of an average adult. The manikin was equipped with five different commercially available masks that have seen widespread use throughout the course of the COVID-19 pandemic. Both near-field flow visualizations and far-field particle concentration measurements allow for a holistic investigation of the effect of masks and ventilation in the test room, and provide a quantitative measure relative to aerosol concentrations and mask efficiencies of interest for transmission risk assessment, model development, and implementation of adaptive health and safety practices at workplace. The results highlight that (i) considerable relative aerosol concentration levels can be reached at a 2 m distance from the subject in an unventilated space, and even when the subject is equipped with a mask, the relative concentrations are notably higher than those expected based on the ideal/rated efficiency of the masks; (ii) fit of the mask to the face, in terms of limiting leakage around the mask perimeter, is critical for limiting aerosol dispersion in an unventilated space, especially for high efficiency masks (e.g., N95/KN95); and (iii) increased ventilation/air-cleaning capacity significantly reduces the transmission risk in an indoor environment, surpassing the apparent mask filtration efficacy even at relatively low air-change rates ( ∼2 room volumes per hour).

The baseline filtration characteristics for the various masks tested in this study indicate that more than 50% of aerosols (polydisperse, 1 *μ*m mean diameter) can pass through the material of commercially available cloth and surgical masks in ideal conditions (zero leakage due to fit), whereas ideal filtration efficiency is 95% (or higher) in the case of KN95 and R95 masks. Flow visualizations and velocity measurements in the near-field (immediate vicinity of the face) indicate that none of the tested masks is performing at their ideal filtration efficiencies due to leakages through gaps in the fit of the mask. This occurs around the cheeks, below the jaw, and at the bridge of the nose, with the latter being the most significant for all masks. Aerosols are seen to escape through these leakage sites in the form of concentrated particle clouds that do not mix quickly with the ambient air on account of relatively low flow velocities and hence low levels of turbulent mixing. The degree of leakage varies between masks, with high-efficiency masks, such as the KN95, performing better. Factors affecting leakage at the mask perimeter include mask geometry, strap style and elasticity, and whether or not the mask is equipped with a deformable nose piece that can be tightly shaped to the nose. Furthermore, although the present study does not characterize the effectiveness of masks during inhalation, the aforementioned loss of filtration efficiency due to perimeter leakage is also expected to be present during inhalation, although it is to a lesser extent due to the improved sealing effect produced by the negative pressure difference relation to the ambient.

The near-field velocity measurements indicate that the forward momentum of breath exhaled through the nose is reduced significantly and redirected when the subject is equipped with a mask. Furthermore, this attenuation of the forward momentum increases with the filtration efficiency of the mask material when a proper fit is ensured. Thus, the present results endorse the use of high-efficiency, unvalved masks with a proper fit when the recommended social distancing guidelines cannot be maintained between individuals.

Measurements of aerosol concentration at a 2 m distance from the subject show a characteristic increase in average particle concentration with time in the absence of ventilation, following the first order response based on the well-mixed room model. Across all cases, relative particle concentrations saturate at elevated levels, indicating accumulation of aerosol particles within the room. When the subject is not fitted with a mask, the saturation concentration is the highest among all the cases tested. A decrease in saturation concentration is seen for all mask types; however, the effective filtration is notably lower than the ideal filtration efficiency of the material due to leakages in accordance with a mask's ability to decrease the number of particles released into the room per breath. Thus, the apparent filtration efficiency of a mask ( $\eta_{\text{AFE}}$) is estimated based on the relative difference in saturation concentration at the measurement location between cases with and without a mask. This metric provides a more representative measure of mask efficiency and is of particular interest for future modeling studies and continuous occupancy risk assessment.

The results show that a standard surgical and three-ply cloth masks, which see current widespread use, filter at apparent efficiencies of only 12.4% and 9.8%, respectively. Apparent efficiencies of 46.3% and 60.2% are found for KN95 and R95 masks, respectively, which are still notably lower than the verified 95% rated ideal efficiencies. Furthermore, the efficiencies of a loose-fitting KN95 and a KN95 mask equipped with a one-way valve were evaluated, showing that a one-way valve reduces the mask's apparent efficiency by more than half (down to 20.3%), while a loose-fitting KN95 provides a negligible apparent filtration efficiency (3.4%). The present results provide an important practical contrast to many other previous experimental and numerical investigations, which do not consider the effect of mask fit when locally evaluating mask efficiency or incorporating mask usage in a numerical model. Nevertheless, if worn correctly, high-efficiency masks still offer significantly improved filtration efficiencies (apparent and ideal) over the more commonly used surgical and cloth masks, and hence are the recommended choice in mitigating the transmission risks of COVID-19.

The directivity of aerosol dispersion was assessed through concentration measurements at a 2 m distance and at locations in front of (0°), to the side of (90°), and behind (180°) the subject with a surgical and KN95 masks. For all the cases, the effect of orientation was less than about 10% of the local particle concentration and indicated a relatively minor directivity effect at a distance of 2 m. It is conjectured based on the flow measurements in the vicinity of the manikin face that significant directivity effects are confined to the relatively close proximity of the host.

The effect of ventilation/air-cleaning was consider using a HEPA air purifier at the recommended pre-pandemic air-change rates (ACH = 1.7– $3.2\;h^{-1}$). The results show that ventilation air-exchange or purification is effective in decreasing both the final saturation concentration and the time required to reach the saturation state. Based on the apparent filtration efficiency, tests performed with no mask at an air-change rate of $1.7\;h^{-1}$ (and higher) outperform cases with high-efficiency masks (KN95 and R95) and no room ventilation. However, at these low ventilation rates, a notable particle concentration is still present at a 2 m distance, which is indicative of higher ventilation rates needed to ensure negligible aerosol build-up over prolonged occupancy.

## Data Availability

The data that support the findings of this study are available from the corresponding author upon reasonable request.
